# Operationalization of the Clinical Frailty Scale in Korean Community-Dwelling Older People

**DOI:** 10.3389/fmed.2022.880511

**Published:** 2022-06-10

**Authors:** Hee-Won Jung, Ji Yeon Baek, Il-Young Jang, Eunju Lee

**Affiliations:** Division of Geriatrics, Department of Internal Medicine, Asan Medical Center, University of Ulsan College of Medicine, Seoul, South Korea

**Keywords:** clinical frailty scale, culture, classification, Asian, older adults

## Abstract

**Background:**

The Clinical Frailty Scale (CFS) is a simple measure of global fitness validated in various populations in real-world settings. In this study, we aimed to assess the characteristics and validities of the CFS in community-dwelling older people in Korea, with the original classification tree (oCFS) and a culturally modified tree (mCFS).

**Methods:**

The comprehensive geriatric assessment records of 1,064 individuals of the Aging Study of the Pyeongchang Rural Area were used for this study. For mCFS, we considered the dependency of the food preparations and household chores not to be deficits in the male population. The frailty index was used as a reference for construct validity. We used a composite outcome of death and institutionalization for outcome validity.

**Results:**

The correlation coefficients with frailty index were higher in mCFS (.535) than in oCFS (.468). The mean frailty index was lower in individuals reclassified by mCFS (5 to 4) than people who stayed in mCFS 5. The classification coefficient of mCFS was significantly higher than that of oCFS (*p* <0.001) in determining people with frailty (frailty index.25 or higher). Trends of a higher incidence of the composite outcome were observed in both higher oCFS and mCFS, in which oCFS and mCFS did not differ significantly in predicting the risk of the outcome.

**Conclusion:**

The classification tree of CFS could be culturally adopted in a community-dwelling population of Korea and considered valid in detecting the vulnerable population.

## Introduction

Korea is experiencing the fastest pace of transformation in the population structure among developed countries and facing social and economic challenges from its aging population (1). Accompanied by a rapid shift in social structure, including the decreasing family size and urbanization, Korea is expecting decades of a super-aged society. Meanwhile, even Western countries with better resources and a slow rate of population aging have been struggling to maintain high-quality services for their older populations (1, 2). The long-term care system in Korea is expected to be overwhelmed in the near future by soaring care demands from older adults with disabilities (3). Hence, there is an urgent need to develop sustainable care models to address the spectrum of individual care demands while optimizing available resources.

Growing knowledge on the importance of the frailty spectrum might be one of the most remarkable achievements in geriatric medicine in this century in person-centered risk assessment and care provision for older adults (4–6). Measured by either the phenotype or the index, the spectrum of frailty has shown high prediction ability for adverse health outcomes encompassing healthcare use and incidence of geriatric syndromes, such as fall and delirium, as well as disability and death (7, 8). These features of frailty have led to a growing research interest in incorporating frailty as a risk measure in many specialized fields of medicine outside geriatrics (9). Translation studies have shown content validity of the frailty spectrum as a measure of human biological age (10, 11). It has been reported that frailty was a dynamic and malleable feature, which responded to appropriately designed multicomponent intervention targeting geriatric functional domains (12, 13). Consequently, addressing the frailty spectrum might be a potential cornerstone in designing tailored care models for the older population, encompassing areas of health promotion, disease treatment, prevention of disabilities, and even long-term caregiving.

Despite its importance, assessing and interpreting frailty has long been regarded as a barrier in incorporating this condition into real-world clinical practice (14). However, the Clinical Frailty scale (CFS), a simplified measure including pictographs and brief descriptions on global fitness and functional capabilities, has been suggested as a viable approach (15, 16). After being initially developed to trace the frailty index, validity of the CFS has been shown in diverse spectrums of care situations, including intensive care units, long-term care facilities, and sometimes in a large population (15, 17). In the Korean population, studies have shown validities of the CFS in geriatric outpatients and inpatients in a hospitalist unit and an intensive care unit (18–20). However, to the authors' knowledge, its utility and validity in community-dwelling older people in Korea are yet to be proven, even though the CFS has potential advantages as an efficient measure of the frailty in community-based public health programs. Furthermore, in previous attempts adopting CFS in the present authors' institution, we faced questions on interpreting and classifying capabilities on instrumental activities of daily living (IADL) of male patients.

Recognizing this knowledge gap, we aimed to assess the characteristics and validities of the CFS in community-dwelling older people in Korea. We measured the CFS by adopting the decision tree, which was recently developed and validated by Theou et al. (21), and assessed its characteristics with the generally well-accepted measures of frailty phenotype and frailty index. In this adoption, we compared the original classification and culturally modified classification considering controversial IADL items of older male population in Korea. We then evaluated whether the CFS could predict a composite outcome of institutionalization and death.

## Methods

### Study Design and Population

We used the records of the Aging Study of the Pyeongchang Rural Area (ASPRA), a population-based prospective cohort study on frailty, sarcopenia, and geriatric syndromes in community-dwelling older Korean adults. Details of the study characteristics, evolutions in the designs, and summary of study findings have been described previously (22). Briefly, the ASPRA was established in 2014 in Pyeongchang county, Gangwon Province, Korea, using the Community Health Post network of the National Healthcare Service (NHS), a health system operated by the Korean government. It started with 382 individuals in three small villages and gradually expanded to the surrounding regions, eventually including 1,529 participants who underwent at least one examination before December 2018.

In the study, we used the records of the 1,064 participants who were recruited and underwent baseline examination from July 2015 to June 2018, including the Comprehensive Geriatric Assessments (CGA) assessing frailty, disability, and were assessed for a composite outcome as described below. As the International Physical Activity Questionnaire Short Form (IPAQ) included an item on the “participation of strenuous activity” used in the classification tree for CFS, which was introduced in 2015, we excluded 382 participants who had assessments from October 2014 to June 2015 without IPAQ.

The eligible participants in the ASPRA were individuals aged 65 years or older, living at home, able to walk with or without assistive devices, able to provide informed consent by themselves or their legal proxy, and registered in the NHS. Persons living in nursing homes, chronic care hospitals, or receiving nursing-home level care at home due to disabilities were excluded. Also, individuals apparently approaching their end-of-life were not considered to be eligible. The protocol of this study was approved by the Institutional Review Board of Asan Medical Center. Written informed consent was obtained from all participants or their legal proxy.

### Classification of the CFS

The original classification of the CFS (oCFS) was scored using the classification tree (Supplementary Figure 1) adapted to be compatible with variables of the ASPRA from previous work by Theou et al. that showed good agreement with an expert rated CFS (21). The classification tree was developed based on the descriptions of individual CFS levels and included basic activities of daily living (ADL) and IADL, chronic conditions, self-rated health, energy level, and physical activity.

We used parameters from the baseline CGA in the study to capture variables in the classification tree. For ADL, we used items on dressing, bathing, eating, walking, and transferring from the Korean ADL (23). The IADL included items such as using a phone, shopping, food preparation, household chores, managing medications, and handling own money from the Korean IADL (23). Chronic conditions included 11 physician-diagnosed illnesses, such as angina, arthritis, asthma, cancer, chronic lung disease, congestive heart failure, diabetes, heart attack, hypertension, kidney disease, and stroke. Self-rated health was measured as excellent, good, or poor. Poor endurance and energy (everything is an effort) were assessed by the exhaustion item used in the Cardiovascular Health Study (CHS) frailty phenotype described below (24). Engagement in strenuous sport or recreational activities was captured using the IPAQ questionnaire (25).

The cultural characteristic of the Korean older family has long been considered an issue in assessing disability in IADL items with the presence of gender-specific differences in household-related items (26). For the culturally modified classification of the CFS (mCFS), we excluded male participants with dependencies in IADL only for items on preparing meals and doing housework from the determination of CFS 5.

### Frailty Index and Phenotype

We used a 34-item frailty index as a standard measure of frailty in this study (Supplementary Table 2). The frailty index ranging from 0 to 1 using the deficit accumulation approach was established from 34 health-related items encompassing the domains of chronic conditions, physical performance, cognitive function, and daily functions (7, 27, 28). We established cut-off values for the frailty index adopting definitions and observations of previous studies (7, 29), and a frailty index of 0.25 or higher as frail.

As a measure of the physical frailty, CHS frailty phenotype comprised the following items: (1) exhaustion (“moderate or most of the time during the past week” for either of “I felt that everything I did was an effort” or “I could not get going”); (2) low activity (lowest 20 percentile in physical activity using the IPAQ); (3) slowness (usual gait speed <0.8 m/s in the 4-m walk); (4) weakness (dominant hand grip strength <26 kg for men and <17 kg for women); and (5) weight loss (unintentional weight loss > 3 kg during the previous 6 months). We considered a CHS frailty phenotype score of 3 or higher as indicating frailty (24).

### Co-variables

Basic demographic, anthropometric, and social information, including the education level, were recorded by the interviewers. Geriatric functional parameters were assessed from the CGA performed by the trained nurses. We considered the disability in ADL as the presence of dependency in at least one in seven items, such as bathing, continence, dressing, eating, toileting, transferring, and washing the face and hands, in the Korean ADL (23). Similarly, the disability in IADL was determined as the presence of at least one dependency in 10 items: food preparation, household chores, going out a short distance, grooming, handling finances, doing laundry, managing medications, shopping, transportation, and using a phone, in the Korean IADL (23). Cognitive dysfunction was determined with the Korean version of the Mini-Mental State Examination (K-MMSE) score cut-off of <24 (30). From the medication history, we defined polypharmacy as the use of five or more prescription medications. History of fall in the previous 12 months was recorded.

### Outcome Assessment

We used a composite endpoint of death and long-term institutionalization due to functional impairment as an outcome. This information was acquired by telephone interviews with the participants or their family members, performed every 3 months. Death was additionally captured from records of the Community Health Post network system. For this analysis, we used composite outcome data captured until August 2020.

### Statistical Analysis

Descriptive characteristics according to oCFS and mCFS were calculated. Spearman's correlation coefficients between oCFS, mCFS, and frailty index, and CHS frailty phenotype score were calculated. Boxplots were used to display distributions of frailty index upon each score of oCFS and mCFS. The receiver operating characteristics (ROC) analysis, net classification improvement (NRI), and integrated discrimination improvement (IDI) were used to compare the classification ability of oCFS and mCFS for frailty index 0.25 or higher. Kaplan–Meier and Cox regression analysis were used to evaluate the impact of oCFS and mCFS on the composite outcome. Before performing the Cox regression, the proportional-hazards assumption was checked using the log-log plots. In Cox regression analyses, age and sex were introduced as covariables in Model 2, and the number of chronic conditions was further included in Model 3, in which the CFS 1 was considered a reference. The discriminatory ability was assessed using the Harrell's C index (31), and compared using linear comparison. Two-sided *p*-values of <0.05 were considered significant. Statistical analyses were performed using the Stata 15.0 (StataCorp, College Station, TX, USA).

## Results

### Descriptive Characteristics

To compare basic demographic and clinical parameters, we grouped participants into two groups: CFS 1–3 (*n* = 398, 37.4%); and CFS 4–7 (*n* = 666, 62.6%), according to our previous observation suggesting that CFS 4 or higher as a cut-off for the frailty in the geriatric outpatients in Korea. Parameters between the two groups are shown in Table 1. The population in the higher CFS group were older, less educated, had more chronic conditions, and were living with a high frailty index and CHS frailty phenotype score. Individuals in the higher CFS group had a high number of impaired ADL and IADL items, a low MMSE score, and was more likely to experience a fall in the previous year.

**Table 1:** Basic demographic and clinical characteristics.

	**CFS 1-3**	**% or SD**	**CFS 4-7**	**% or SD**	***p*-value**
Sample size (*n*, %)	398	37.4	666	62.6	
Age (mean, SD)	73.6	5.5	77.4	7.1	<0.001
Women (*n*, %)	203	51.0	380	57.1	0.055
Years of education (mean, SD)	7.4	4.1	5.3	3.2	<0.001
Number of chronic conditions (mean, SD)	1.1	1.0	1.5	1.1	<0.001
CHS frailty score (range: 0–5) (mean, SD)	1.0	1.0	1.8	1.2	<0.001
Frailty index (range: 0–1) (mean, SD)	0.11	0.07	0.23	0.14	<0.001
Number of impaired ADL items (mean, SD)	0.07	0.25	0.46	1.64	<0.001
Number of impaired IADL items (mean, SD)	0.02	0.15	2.08	3.82	<0.001
MMSE score (mean, SD)	26.9	3.2	24.4	4.9	<0.001
Number of daily medications (mean, SD)	2.3	2.4	3.3	3.1	<0.001
Falls in the previous 1 year (*n*, %)	33	8.3	104	15.6	0.001

*ADL, activities of daily livings; CFS, Clinical Frailty Scale; CHS, Cardiovascular Health Study; IADL, instrumental activities of daily living; MMSE, Mini-Mental State Examination; SD, standard deviation*.

### Comparisons Between oCFS and mCFS as Frailty Constructs

**Figure 1:**
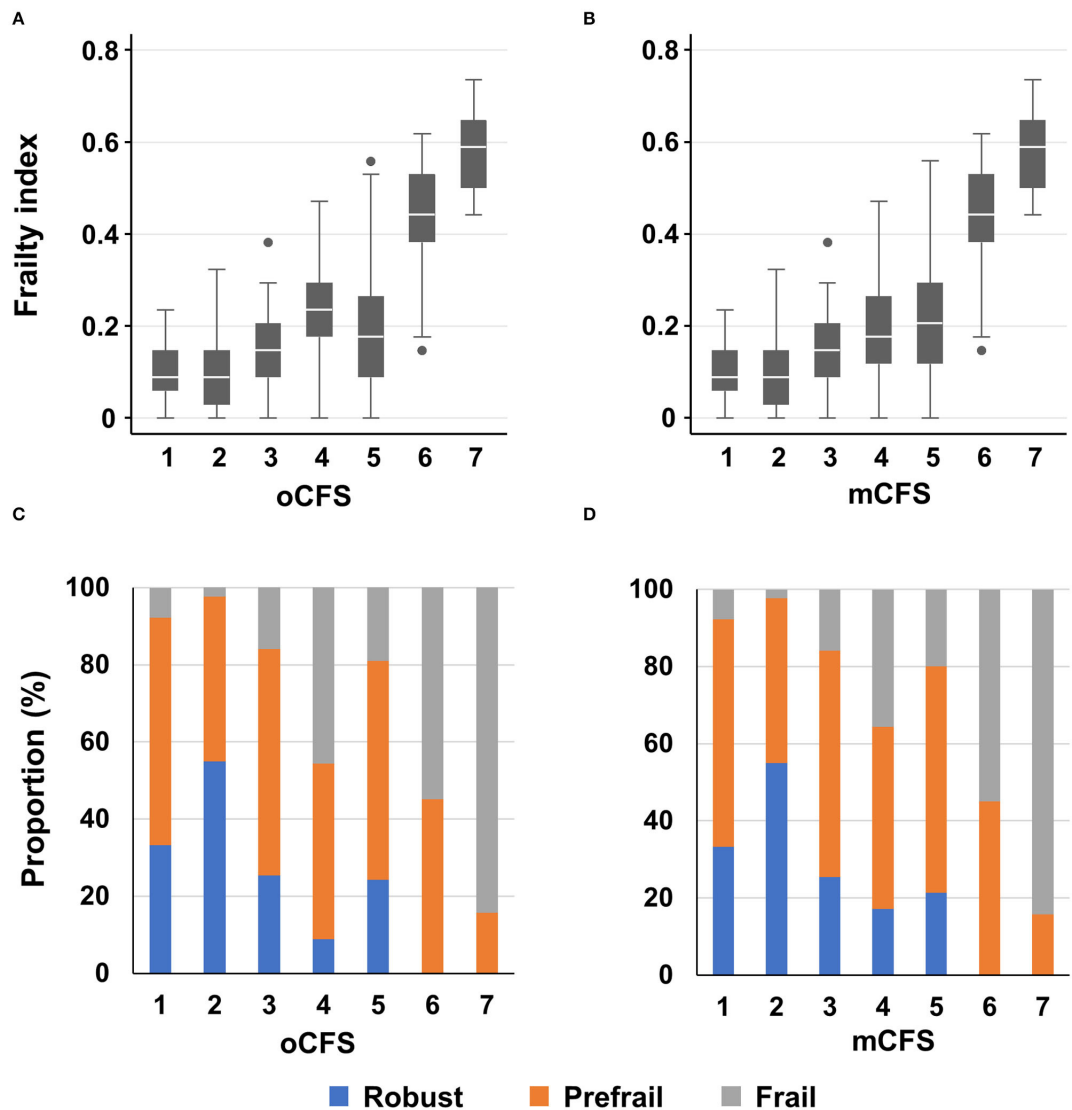
Boxplots for frailty index according to clinical frailty scale scores, by original classification tree [oCFS, **(A)**] and culturally modified classification tree [mCFS, **(B)]**, and bar plots showing the prevalence of the Cardiovascular Health Study frailty phenotype categories according to oCFS **(C)** and mCFS **(D)**; In the box plot, the upper, mid, and lower lines denote 75th, 50th, and 25th percentiles and upper and lower margin of whiskers denote ± 1.5 interquartile range from the 50th percentile. Data outside the ± 1.5 interquartile range from the 50th percentile are shown as outliers.

By oCFS, 89 (8.4%), 171 (16.1%), 138 (13.0%), 193 (18.1%), 403 (37.9%), 51 (4.8%), and 19 (1.8%) individuals had an oCFS score of 1–7, respectively. Among the male population, 98 persons who were initially considered to have an oCFS of 5 had impairments in IADL only for items on preparing meals and doing housework care needs. Hence, 291 (27.4%) and 305 (28.7%) persons were considered to be mCFS 4 and 5. The mean (SD) frailty index was 0.10 (0.06), 0.09 (0.07), 0.14 (0.07), 0.23 (0.09), 0.19 (0.12), 0.43 (0.10), and 0.58 (0.09) for the oCFS scores of 1–7, respectively (*p*-value for the trend of the frailty index by oCFS, <0.001). By mCFS, the mean (SD) frailty index was 0.19 (0.10) and 0.22 (0.12) for mCFS 4 and 5 (*p*-value for the trend of frailty index by mCFS <0.001). Distributions of the frailty index and CHS frailty phenotype categories by groups of oCFS and mCFS are shown in Figure 1.

The correlation coefficients for oCFS and mCFS with frailty index were 0.468 (*p* < 0.001) and 0.535 (*p* < 0.001), respectively. Those for oCFS and mCFS with CHS frailty phenotype score were 0.277 (*p* < 0.001) and 0.320 (*p* < 0.001), respectively. In summary, the correlation coefficients with two frailty measures were higher in mCFS than in oCFS.

We compared the frailty index among male participants in CFS 4–5 to assess the potential impact of reclassification from oCFS to mCFS. The mean (SD) frailty indexes of reclassified individuals were 0.12 (0.08), which was significantly lower (*p* = 0.014 by *t*-test) than those who remained in mCFS 5, of 0.15 (0.11).

To compare the explanation abilities of oCFS and mCFS, we performed an ROC analysis with frailty index as an anchor. The area under the curve of mCFS [0.812, 95% confidence interval (CI): 0.787–0.837] was significantly higher (*p* < 0.001) than that of oCFS (0.776, 95% CI: 0.749–0.803), to classify the frailty that was determined by the frailty index 0.25 or higher. For the accurate comparison of oCFS with mCFS, we additionally calculated the value of NRI and IDI using frailty index (cutoff 0.25) as reference. NRI was 0.193 [standard error (SE) 0.043] (*p* < 0.001), and IDI was 0.071 (SE 0.006) (*p* < 0.001), which means the new model (mCFS) classifies frailty status better than the original model (oCFS) significantly.

### Outcome Relevance of oCFS and mCFS

**Figure 2:**
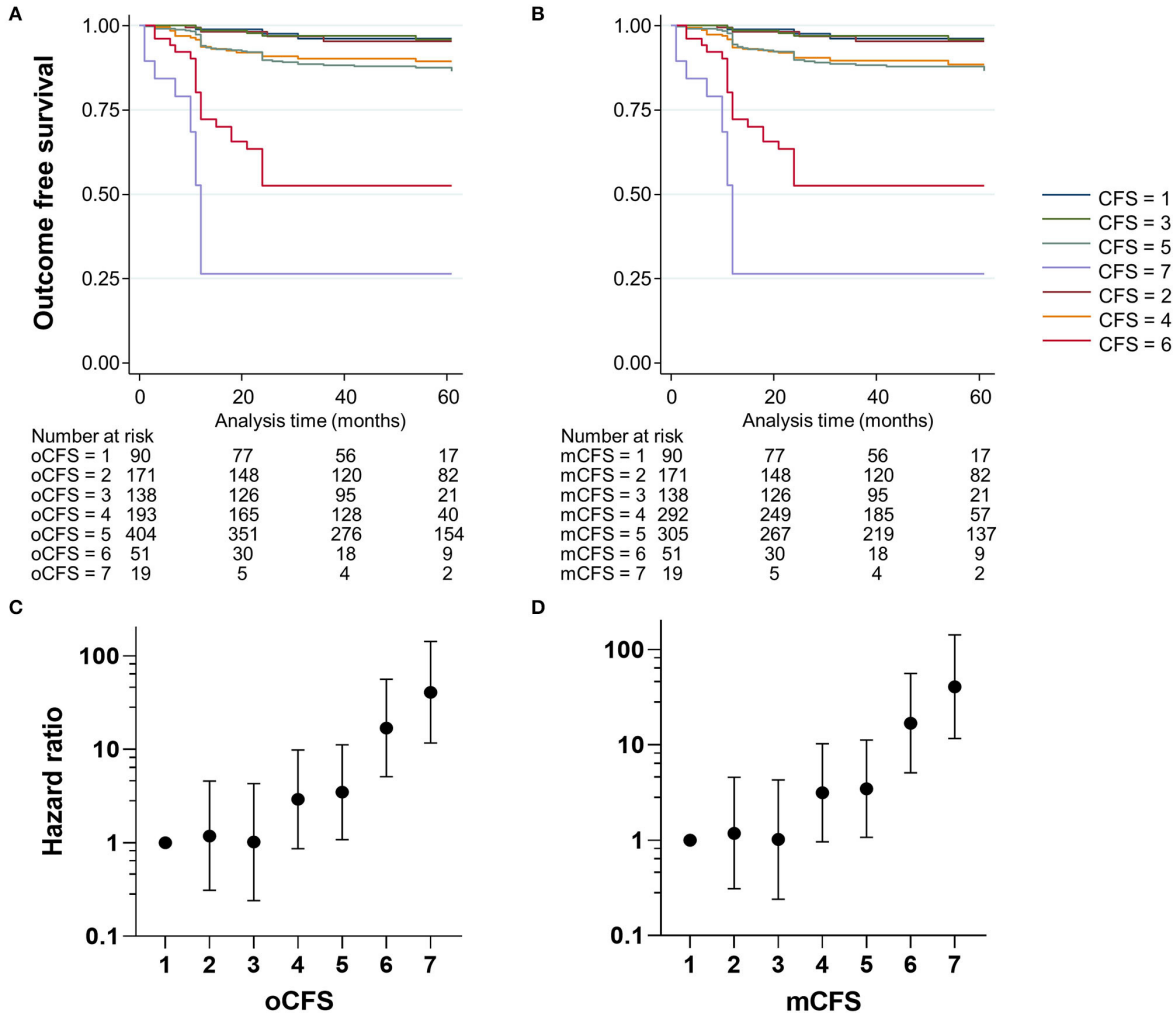
Kaplan–Meier plots showing the incidence of the composite outcome according to clinical frailty scale scores, by original classification tree [oCFS, **(A)**] and culturally modified classification tree [mCFS, **(B)**], and plots showing hazard ratios (dots) and 95% confidence ratio (whiskers) of the composite outcome by oCFS **(C)** and mCFS **(D)**.

To assess the outcome validity of oCFS and mCFS, we performed survival analysis using the data of the composite outcome in the study population. The Kaplan–Meier curves for oCFS and mCFS scores are shown in Figures 2A,B, respectively. Hazard ratio (HR) and 95% CI using univariate for oCFS and mCFS scores are displayed in Figures 2C,D, respectively. The increasing burden of the frailty either by oCFS or mCFS were associated with a higher risk of composite outcome incidence, and statistical significances were maintained after adjusting for age, sex, and the number of chronic conditions (Table 2). The crude incidence rate of composite outcome and HR of each CFS score is shown in Supplementary Table 1.

**Table 2:** Increased risk of composite outcome according to Clinical Frailty Scale scores, by original classification tree (oCFS) and culturally modified classification tree (mCFS).

	**Model 1**		**Model 2**		**Model 3**	
	**HR**	**95% CI**	**HR**	**95% CI**	**HR**	**95% CI**
**oCFS (by 1 increment)**	2.08	1.75–2.47	1.64	1.38–1.95	1.59	1.34–1.90
Age (by 1-year increment)			1.11	1.08–1.14	1.11	1.09–1.14
Sex (ref, male)			0.63	0.44–0.91	0.57	0.39–0.83
Number of chronic conditions (by 1 increment)					1.19	1.01–1.40
**mCFS (by 1 increment)**	2.04	1.74–2.41	1.63	1.37–1.93	1.58	1.33–1.87
Age (by 1-year increment)			1.11	1.08–1.14	1.11	1.08–1.14
Sex (ref, male)			0.58	0.41–0.84	0.53	0.36–0.77
Number of chronic conditions (by 1 increment)					1.20	1.02–1.41

*Model 1: crude model; Model 2: adjusted for age and sex; Model 3: adjusted for age, sex, and number of chronic conditions*.

*CI, confidence interval; HR, hazard ratio*.

Prediction performances for the composite outcome of oCFS and mCFS were compared with the frailty index. The Harrell's C statistics were 0.771 (95% CI: 0.727–0.815), 0.721 (95% CI: 0.675–0.768), 0.719 (95% CI: 0.671–0.766) for the frailty index, oCFS, and mCFS, respectively. There was no significant difference between the C statistics of oCFS and mCFS (*p* = 0.719). However, the C statistics of both oCFS (*p* = 0.016) and mCFS (*p* = 0.004) were significantly lower when compared with that of the frailty index.

## Discussion

In this study, we evaluated the validities of oCFS and mCFS in community-dwelling Korean older adults, using the classification tree of CFS that was previously validated in a population from the United Kingdom. When the frailty index was used as a standard, mCFS was better than oCFS in construct validity. On the other hand, mCFS and oCFS were comparable in predicting the composite outcome. To the authors' knowledge, this is the first study adopting the classification tree of CFS in an Asian population evaluating construct and criterion validity.

The growing consensus support that frailty is a core clinical feature of the complex system of aging physiology (5), and both the phenotype model and the deficit-accumulation model converge with each other (7), even though controversies still exist to date on the biological and clinical construct of frailty. The CFS was conceptualized from a theoretical model of fitness and frailty (32), encompassing the functional spectrum as a measure of global fitness (16). In the original study, the CFS highly correlated with the frailty index and had comparable prediction ability for outcomes of mortality and institutionalization (16). Hence, the validity of the CFS from the classification tree is predictable because of its high agreement with the CFS by the geriatricians (21). Even though the CFS directly measured by the geriatricians were unavailable, the CFSs determined from the CGA records correlated with both the frailty index and CHS frailty phenotype, supporting convergent validity of the classification tree.

For mCFS, we did not consider the presence of dependency in IADL items of household chores and preparing meals as sufficient to classify as CFS 5 in men. Controversies existed in interpreting care needs in IADL items in the older Asian populations in geriatrics and gerontology research (26, 33), as traditionally, household work was largely performed by women in the region. In a Korean study on gender differences of ADL and IADL items, men were more likely to rate themselves as dependent in household activities, such as preparing meals and doing laundry (26). A similar gender bias was noted in a Singaporean study, reporting high dependency in preparing meals, doing laundry, and taking medication in men (34). Despite the relevance of these items in the clinical construct of IADL disability being difficult to conclude, we observed that the convergent validity of the CFS was better when dependencies in household items were interpreted leniently (mCFS) than strictly (oCFS) in men. Furthermore, the frailty index of men reclassified from CFS 5 to CFS 4 by the 'cultural modification' was lower than the population who remained at CFS 5. These findings support the potential cultural impact has on defining the construct of disability and frailty, calling for research on this topic as many Asian countries are experiencing rapid population aging and the need to establish social support systems for their aging population (2).

There was no significant difference between mCFS and oCFA in predicting the composite outcome of death and institutionalization. As HRs of CFS 4 and CFS 5 for both classifications were relatively similar, the effect of the reclassification would be minimal in prediction ability for the outcome. As we merged the two outcomes of death and institutionalization into a composite outcome in this analysis, given limitations in sample size and observation period, we could not dissect the impact of IADL interpretation on mortality and functional decline. The minimal outcome differences between CFS 4 and CFS 5 might be due to availability of long-term care insurance that provides at-home assistance for community-dwelling older adults with disabilities in IADL items. Yet, the impact of this service on preventing institutionalization of people living with IADL impairment cannot be proven under the current study design (3). However, with survival analysis showing a discrete and decisive increment in outcome risk from CFS 4, the CFS could be used to screen community-dwelling older adults at risk of adverse health outcomes.

Evidence supports well-designed multicomponent intervention programs that effectively prevent adverse health outcomes in community-dwelling older adults (12, 35, 36). A recent paper published from Korea, analyzing the long-term outcomes of a 6-month program with a propensity-score-matched control group, showed that both the frailty status and physical performance was better in the intervention group, and the benefit in physical performance remained significant for 2 years after the end of the program (12). Furthermore, the 30-month mean institutionalization-free survival time was longer by 5.2 months in the intervention group than in the comparison group. To scale up these programs with multiple domains in the public health scale for a more extended period, selecting the target population that might benefit maximally from such interventions is necessary. In this regard, early programs may start by focusing on the population of CFS 4–5, considering its prediction ability for institutionalization-free survival. Given the brief nature of the CFS, the burden of finding mass-scale cases might be minimal.

There are several limitations to this study. We used recorded data of the CGA to estimate oCFS and mCFS, rather than prospectively measuring CFS by geriatricians. Nevertheless, as we constructed the CFS from individual items of the CGA, we were able to tag individuals from IADL items; however, recording all relevant IADL items and frailty index parameters might be less feasible in real-world CFS examinations by geriatricians. Generalizability is limited, as we were only able to assess cultural impact in determining the CFS in a community-dwelling population in a rural area in Korea. As noted in the literature, cultural characteristics are rapidly changing with alterations in family and social structures in Asian countries, including China (37). Our data were acquired from a rural area in the mid-2010s, and the results might differ if performed using individuals in an urban area. Also, baseline characteristics of participants with or without IPAQ differed considerably in terms of age, frailty, and other geriatric parameters (Supplementary Table 3), which might act as a limitation for generalizability. As the current analysis was from the observational cohort, we could not assess the potential effect of interventions on the CFS, as studies showing the frailty spectrum could be altered by interventions (12). As the ASPRA primarily comprised of community-dwelling, ambulatory participants, we had no CFS 8–9 individuals.

In conclusion, the classification tree of CFS could be culturally adopted in a community-dwelling population in Korea and considered valid in detecting vulnerable population. As such, further studies are warranted to assess the feasibilities and benefits of CFS when performed in the public health scale to screen vulnerable population who might benefit from community-based programs.

## Data Availability Statement

The raw data supporting the conclusions of this article will be made available by the authors, without undue reservation.

## Ethics Statement

The studies involving human participants were reviewed and approved by Institutional Review Board of Asan Medical Center. The patients/participants provided their written informed consent to participate in this study.

## Author Contributions

H-WJ, JB, I-YJ, and EL conceptualized the study and reviewed and edited the manuscript. H-WJ did the formal data analysis. H-WJ, JB, and I-YJ did the investigation. H-WJ and JB wrote the first draft of the manuscript. H-WJ and I-YJ supervised the study and acquired study funding.

## Funding

This Aging Study of Pyeongchang Rural Area was funded by the Pyeongchang Health Center, Pyeongchang County, Gangwon Province, South Korea. This study was also supported by a grant of the Korea Health Technology R&D Project through the Korea Health Industry Development Institute (KHIDI), funded by the Ministry of Health and Welfare, Republic of Korea (Grant No. HI18C2383), and the Asan Multidisciplinary Committee for Seniors.

## Conflict of Interest

H-WJ cofounded Dyphi Inc., a startup company based on sensor technology. The remaining authors declare that the research was conducted in the absence of any commercial or financial relationships that could be construed as a potential conflict of interest.

## Publisher's Note

All claims expressed in this article are solely those of the authors and do not necessarily represent those of their affiliated organizations, or those of the publisher, the editors and the reviewers. Any product that may be evaluated in this article, or claim that may be made by its manufacturer, is not guaranteed or endorsed by the publisher.
