# Supplementary material for: Operationalization of the Clinical Frailty Scale in Korean Community-Dwelling Older People
Source: Front Med (Lausanne). 2022 Jun 10;9:880511. doi: 10.3389/fmed.2022.880511 (PMC9226398; doi:10.3389/fmed.2022.880511)

Supplementary Material

Supplementary Table 1. Crude incidence rate and hazards of each Clinical Frailty Scale (CFS) score for the composite outcome.

	n (rate per 1,000 py)	Model 1		Model 2		Model 3	
		HR	95% CI	HR	95% CI	HR	95% CI
**oCFS**							
1 (ref)	9.5						
2	10.5	1.18	0.31–4.58	1.23	0.32–4.78	1.23	0.32–4.75
3	9.5	1.02	0.24–4.28	1.02	0.24–4.27	0.98	0.24–4.12
4	27.1	3.15	0.96–10.30	2.54	0.75–8.60	2.34	0.69–7.95
5	31.9	3.46	1.07–11.21	2.56	0.80–8.26	2.42	0.75–7.80
6	178.3	16.83	5.05–56.07	6.72	0.19–23.20	6.08	1.76–21.06
7	440.9	40.77	11.69–142.16	18.63	5.22–66.46	15.6	4.29–56.74
**mCFS**							
1 (ref)	9.5						
2	10.5	1.17	0.30–4.52	1.24	0.32–4.79	1.23	0.32–4.76
3	9.5	1.01	0.24–1.23	1.02	0.24–4.27	0.98	0.24–4312
4	29.8	3.12	0.96–10.22	2.75	0.84–8.99	2.54	0.77–8.36
5	31.0	3.42	1.05–11.08	5.39	0.73–7.82	2.27	0.69–7.43
6	178.3	16.63	4.99–55.40	6.60	1.91–22.84	6.00	1.73–20.82
7	440.9	40.28	11.55–140.46	18.37	5.14–65.59	15.49	4.26–56.33

Model 1: crude model; Model

2: adjusted for age and sex; Model 3: adjusted for age, sex, and number of chronic conditions

CI, confidence interval; HR, hazard ratio; mCFS, culturally modified classification of the CFS; oCFS, original classification of the CFS; py, person-year

Supplementary Table 2. The 34-item frailty index.

Changes in everyday activities	Tiredness all the time
Problems getting dressed	Depression
Problems with bathing	Memory changes
Problems carrying out personal grooming	Changes in general mental functioning
Urinary incontinence	History relevant to cognitive impairment or loss
Toileting problems	History of stroke
Gastrointestinal problems	History of diabetes mellitus
Problems cooking	Arterial hypertension
Problems going out alone	Cardiac problems
Impaired mobility	Myocardial infarction
Musculoskeletal problems	Congestive heart failure
Bradykinesia of the limbs	Lung problems
Poor muscle tone in limbs	Respiratory problems
Poor limb coordination	Malignant disease
Poor coordination, trunk	Other medical history
Poor standing posture	
Irregular gait pattern	
Falls	
Mood problems	

Supplement Table 3. Baseline characteristics in participants of the Aging Study of Pyeongchang Rural Area, with and without IPAQ.

Characteristics	With IPAQ (n=1064)	% or SD	Without IPAQ (n=382)	% or SD	p-value
Age	76.0	6.8	77.3	6.4	0.001
Women (n, %)	583	54.8	219	57.3	0.392
Years of education (mean, SD)	6.1	3.7	6.3	4.0	0.313
CHS frailty score (range: 0–5) (mean, SD)	1.52	1.22	1.68	1.36	0.039
34-item frailty index (range: 0–1) (mean, SD)	0.19	0.13	0.21	0.11	<0.001
ADL disability (n, %)	148	13.9	58	15.2	0.541
IADL disability (n, %)	354	33.3	105	27.5	0.037
Cognitive dysfunction (n, %)	281	26.4	127	33.8	0.006
Polypharmacy (yes) (n, %)	280	26.3	47	12.3	<0.001
Falls in the previous 1 year (n, %)	137	12.9	65	17.0	0.045

Supplementary Figure 1. Classification tree of the original Clinical Frailty Scale.

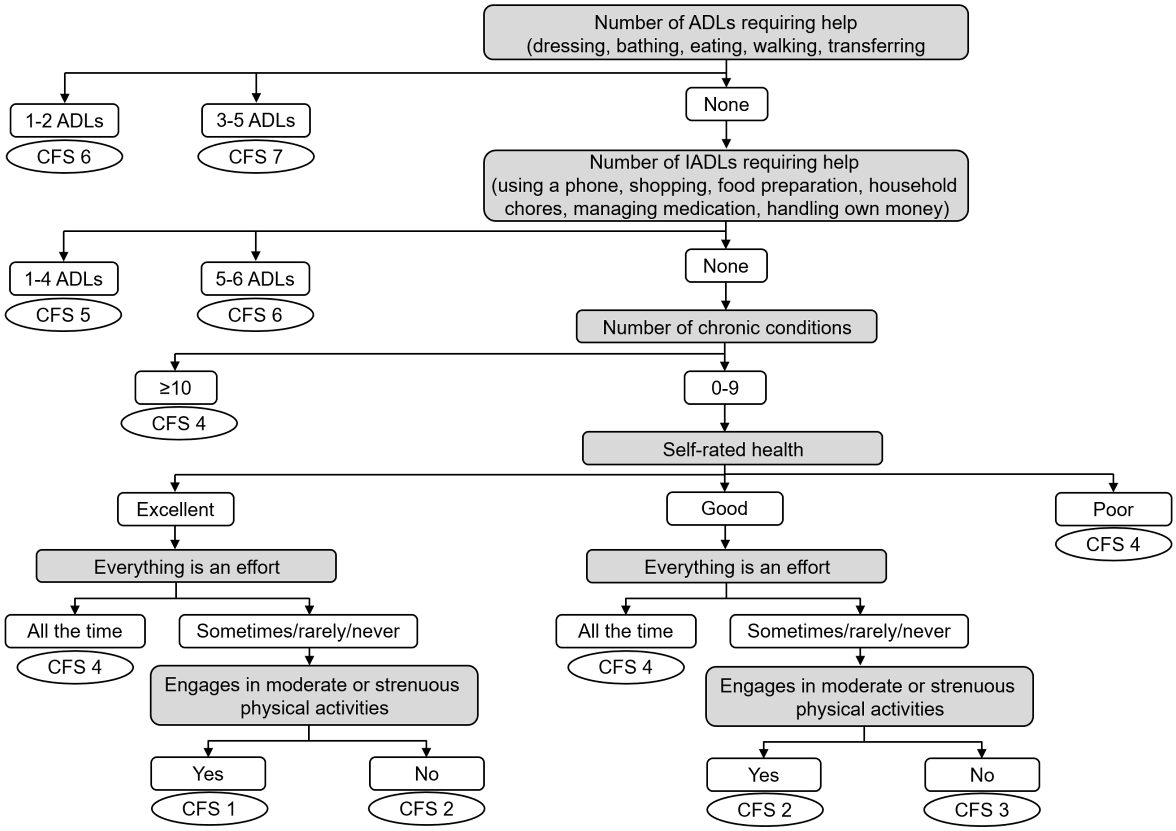

Supplement: Supplementary file 1 [file Data_Sheet_1.pdf]
